# Circular RNA circ-LRP6 facilitates Myc-driven tumorigenesis in esophageal squamous cell cancer

**DOI:** 10.1080/21655979.2020.1809922

**Published:** 2020-08-31

**Authors:** Jiayang Wang, Weiguo Zhu, Guangzhou Tao, Wanwei Wang

**Affiliations:** Department of Radiation Oncology, The Affiliated Huaian No.1 People’s Hospital of Nanjing Medical University, Huai’an, Jiangsu, China

**Keywords:** Esophageal squamous cell cancer, circular RNA, miRNA, Myc, prognosis

## Abstract

Circular RNA (circRNA) circ-LRP6 was recently proven to be a pivotal player in various human diseases. Nevertheless, its role in esophageal squamous cell cancer (ESCC) remains unknown. In the current study, we investigated the expression level, biological function and mechanism of circ-LRP6 in ESCC. Circ-LRP6 was significantly upregulated in ESCC tissues and correlated with malignant clinicopathological characteristics and poor prognosis. Knockdown of circ-LRP6 evidently reduced ESCC cell viability, colony formation and invasion. Circ-LRP6 was mainly located in the cytoplasm and could sponge miR-182 to increase the expression of Myc, a well-documented proto-oncogene. Importantly, circ-LRP6 depletion significantly retarded tumor growth *in vivo*. And silencing of miR-182 or overexpression of Myc effectively rescued the attenuated aggressive phenotype of ESCC cells caused by circ-LRP6 knockdown. Therefore, our data indicate that circ-LRP6 is a novel oncogenic circRNA in ESCC, targeting the circ-LRP6/miR-182/Myc signaling may be a promising therapeutic approach for ESCC patients.

## GRAPHICAL ABSTRACT

**Figure:**
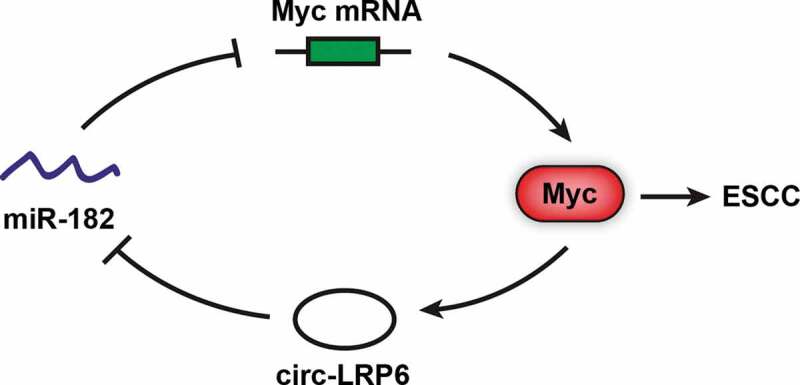

## Introduction

Esophageal squamous cell cancer (ESCC) is the main subtype of esophageal cancer and its morbidity and mortality rank seventh among all malignancies [1]. The current treatments of ESCC mainly include surgical excision, chemotherapy, radiotherapy, interventional therapy and targeted therapy (such as Trastuzumab and Ramucirumab), the therapeutic effect mainly depends on the malignant degree [2]. Patients with ESCC have a poor prognosis, especially those with advanced stage [3]. Therefore, the mechanism underlying ESCC oncogenesis and metastasis needs to be clarified to provide new targets for clinical treatment.

A large number of recent studies have focused on noncoding RNA, especially circular RNA (circRNA), which has a covalently closed ring structure [4]. CircRNA is widespread in eukaryotic cells and is highly evolutionarily conserved [5]. Studies have shown that the expression patterns of circRNA in different tissues, organs, or cells are totally different, hinting that circRNA can be used as a specific disease biomarker [6,7]. Up to now, numerous deregulated circRNAs were identified in human cancers, some of them controlled cancer development and progression through acting as ‘miRNA sponge’, binding to proteins, or translating proteins [8,9]. Recently, a circRNA derived from LRP6 gene (circ-LRP6) were found to be closely correlated with disease progression. For example, Hall et al showed that circ-LRP6 was enriched in vascular smooth muscle cells (VSMC) and affected VSMC migration, proliferation and differentiation [10]. Another study reported that circ-LRP6 was critical for arsenite-induced carcinogenesis [11]. Besides, circ-LRP6 was recently proposed as a tumor-promoting circRNA in osteosarcoma by negatively regulating KLF2 and APC expression [12]. However, an in-depth exploration of the role of circ-LRP6 in ESCC has never been undertaken.

In the present study, we for the first time described the role of circ-LRP6 in ESCC, and found that circ-LRP6 was markedly increased in ESCC tissues as compared with normal tissues. Further investigation revealed that circ-LRP6 promoted ESCC tumorigenesis both *in vitro* and *in vivo* by sponging miR-182 and upregulating Myc oncoprotein.

## Materials and methods

### Ethical Compliance

The clinical and animal experiments of this study were approved by the Ethics Committee and the Institutional Animal Care and Use Committee review board of The Affiliated Huaian No.1 People’s Hospital of Nanjing Medical University.

### ESCC tissues and cell lines

A total of 78 pairs of ESCC and adjacent normal tissues were collected from The Affiliated Huaian No.1 People’s Hospital of Nanjing Medical University. The detailed clinicopathological data are shown in Table 1. We obtained informed consent from all enrolled patients and followed them up. Two ESCC cell lines TE-1 and EC109 were purchased from ATCC and maintained in DMEM medium added with 10% fetal bovine serum and 100 U/mL penicillin-streptomycin.

**Table 1.** The correlation between circ-LRP6 expression and clinicopathological features of patients with ESCC.

		circ-LRP6 expression		
Parameters	All cases (n = 78)	Low (n = 39)	High (n = 39)	*P* value
Gender				
Male	42	23	19	0.364
Female	36	16	20	
Age (years)				
≤ 60	40	18	22	0.497
> 60	38	21	17	
Smoking status				
No	35	18	17	
Yes	43	21	22	0.820
T classification				
T1-T2	45	30	15	0.001
T3-T4	33	9	24	
TNM stage				
I–II	48	31	17	0.001
III–IV	30	8	22	

### Quantitative reverse transcription-PCR (qRT-PCR)

Total RNA was isolated from cultured cells or tissues using TRIzol (Invitrogen, CA, USA) according to the manufacturer’s instructions. The first-strand cDNA was generated by reverse transcription using moloney murine leukemia virus (MMLV) transcriptase (Promega, WI, USA) with random primers. Real-time qRT-PCR was performed on a CFX96 real-time PCR detection system (Bio-Rad, CA, USA). 2^−ΔΔCT^ algorithm was used to determine gene relative expression level.

### Analysis of the subcellular localization of circ-LRP6

For qRT-PCR, cell nucleus/cytoplasm fraction isolation was performed using Nuclear and Cytoplasmic Extraction Kit (Thermo, MA, USA) according to the supplier’s recommendation. GAPDH and U6 are internal reference controls of cytoplasm and nucleus, respectively. For fluorescence in situ hybridization (FISH), Cy3-labeled circ-LRP6 probe were designed and FISH assay was conducted using the FISH kit provided by BerSinBio (Guangzhou, China).

### Lentiviral vector, plasmid and oligonucleotide

Two shRNAs targeting circ-LRP6 were designed and then ligated into pSuper-retro-puro lentiviral vector, followed by infection into TE-1 and EC109 cells. The stable circ-LRP6 knockdown cells were selected by using puromycin. Besides, pcDNA 3.0 Myc-overexpressing plasmid, miR-182 inhibitors and mimics were constructed and transfected into ESCC cells using Lipofectamine 2000 (Invitrogen) following the manufacture’s protocol.

### CCK-8 and colony formation assays

Cell viability assay was performed by seeding 2500 ESCC cells in a 96-well plate, followed by incubation with CCK-8 solution (Dojindo, Kumamoto, Japan). The absorbance at 450 nm was measured using a microplate spectrophotometer (Bio-Rad). For colony formation assay, 500 cells were seeded into a 6-well plate and cultured for 10 days. Then, cells were washed and stained by crystal violet.

### Transwell invasion assay

The upper chamber was coated with Matrigel and then placed at 37°C overnight. Then the unsolidified Matrigel was removed and seeded with 20,000 cells in serum-free DMEM medium. The lower chamber was added with complete culture medium. After 16 h, the trans-well was fixed with 3.7% formaldehyde for 5 min, permeabilized by methanol for 15 min, and then stained by 0.5% crystal violet for 20 min. The cells in the upper chamber (noninvasive cells) were removed and the invaded cells were counted.

### RNA pull-down assay

The biotin-labeled circ-LRP6 probe was designed and synthesized by Sangon (Shanghai, China) to enrich endogenous circ-LRP6 in ESCC cells. Then, 50 pmol above probe was incubated with ESCC cell lysates for 2 h with agitation. The streptavidin magnetic beads (Invitrogen) were added into above complex and incubated for 30 min with agitation. The mixture was washed with 1× wash buffer for five times, then the circ-LRP6-bound miRNAs were eluted by TRIzol and analyzed by qRT-PCR.

### Luciferase reporter assay

ESCC cells were seeded in triplicate into 48-well plates and allowed to settle for 24 h. Then, the wild-type and mutant circ-LRP6 or Myc 3`-UTR luciferase reporters were co-transfected with miR-182 mimics into ESCC cells using Lipofectamine 2000 (Invitrogen). After 48 h, the luciferase activity was tested using a commercial kit (Promega) as per the supplier’s instructions.

### Western blot

The whole cell extract was prepared by lysing cells in NP-40 lysis buffer with proteinase and phosphatase inhibitors (Pierce, CA, USA). Proteins were resolved by 10% SDS-PAGE, then transferred to PVDF membrane and immunoblotted overnight at 4°C with primary monoclonal antibody against Myc (Catalog#MAB3696-SP, R&D Systems, MI, USA) and HRP-conjugated anti-mouse IgG secondary antibody (Catalog#HAF007, R&D Systems). The chemiluminescent signal was developed using Clarity™ Western ECL Substrate (Bio-Rad).

### Animal study

Five nude mice per group were used to ensure the adequate power and each mouse with different weight was randomly allocated. EC109 cells with stable circ-LRP6 knockdown were injected into the armpits of the mice. Then, the mice were maintained under the specific-pathogen-free condition. After five weeks, all mice were sacrificed and the tumor tissues were collected and photographed, and part of the tissues were used for qRT-PCR analysis and western blot assay.

### Statistics

Data were presented as mean ± standard deviation (SD). Student’s t test was used for comparison between two groups. The survival curve of ESCC patients was plotted by Kaplan-Meier method and compared by Log-rank test. All chart making and statistical analysis were performed by using Graph Pad v 7.0 software.

## Results

### Circ-LRP6 is upregulated in ESCC tissues and mainly located in the cytoplasm

**Figure 1.**
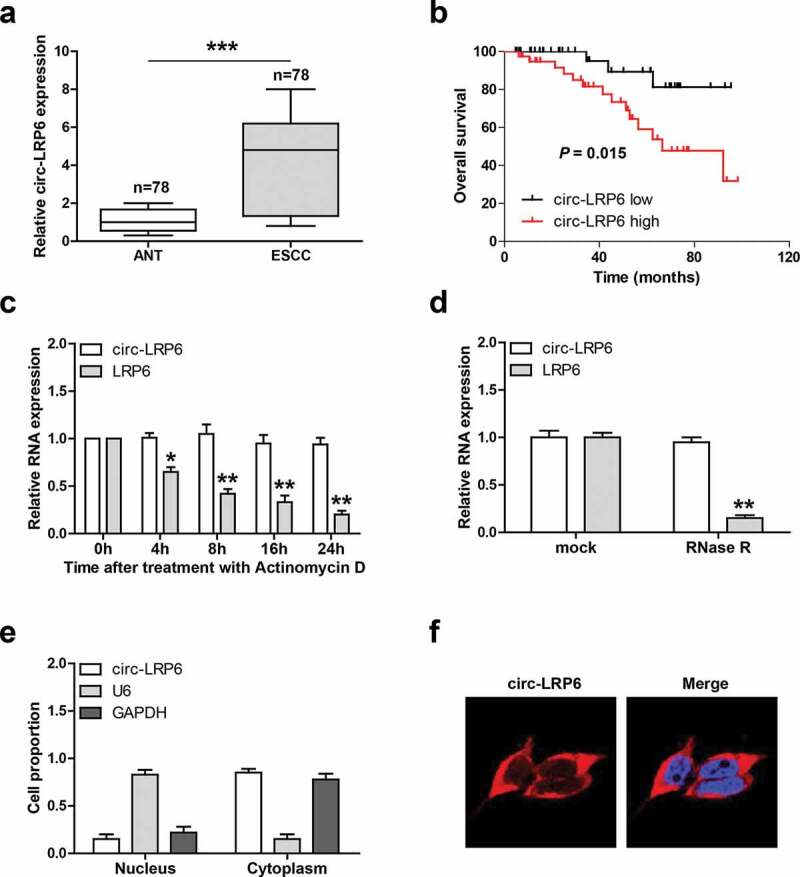
Circ-LRP6 is overexpressed in ESCC tissues. (a) qRT-PCR analysis of circ-LRP6 level in ESCC and normal tissues. (b) The survival curve showing that high circ-LRP6 level predicted poor outcome. (c) qRT-PCR analysis of circ-LRP6 and LRP6 expression at the indicated time after treatment with Actinomycin D. (d) qRT-PCR analysis of circ-LRP6 and LRP6 expression after treatment with RNase R. (e, f) qRT-PCR and FISH assays detecting the location of circ-LRP6. *p < 0.05, **p < 0.01, ***p < 0.001.

We tested the expression level of circ-LRP6 in 78 paired ESCC and normal tissues, as shown in Figure 1(a), the average expression of circ-LRP6 in ESCC tissues was 4.3 times higher than that in normal tissues. And high circ-LRP6 was positively correlated with larger tumor size and later TNM stage (Table 1). Further, ESCC patients with high circ-LRP6 had shorter overall survival time than patients with low circ-LRP6 (Figure 1(b)). Next, we test the half-life of circ-LRP6 by treating cells with Actinomycin D, a transcriptional inhibitor, the qRT-PCR results showed that the half-life of circ-LRP6 was more than 24 h, while that of its linear isoform was less than 8 h (Figure 1(c)). Similarly, circ-LRP6, but not linear LRP6, was highly resistant to RNase R (Figure 1(d)). These suggests that circ-LRP6 is highly stable, which is the nature of circRNA. Lastly, we detested the subcellular localization of circ-LRP6, both qRT-PCR and FISH assays showed that it was preferentially located in the cytoplasm (Figure 1(e,f)).

### Knockdown of circ-LRP6 inhibits ESCC cell proliferation and invasion

**Figure 2.**
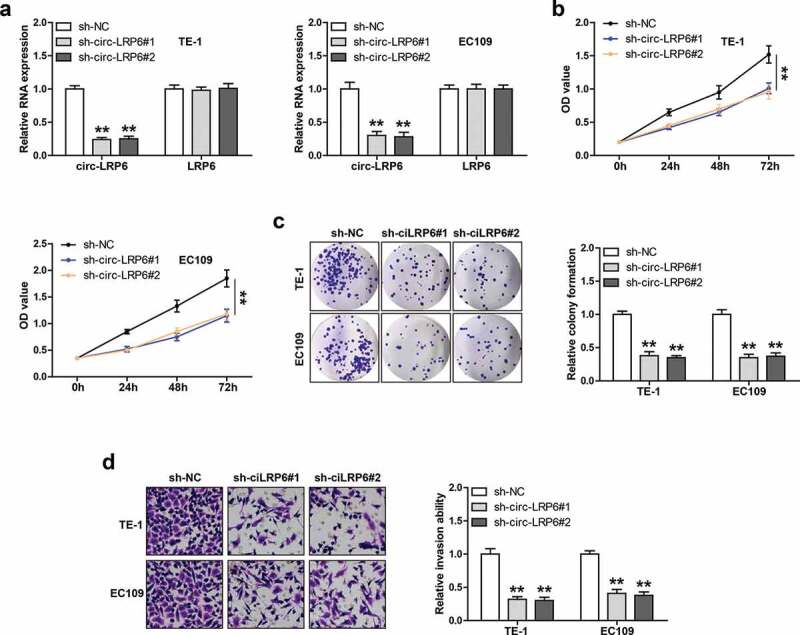
Circ-LRP6 promotes ESCC cell proliferation and invasion. (a) qRT-PCR testing the knockdown efficiency of circ-LRP6 in TE-1 and EC109 cells. (b, c) CCK-8 and colony formation assays detecting the viability and clonal ability of ESCC cells after circ-LRP6 depletion. (d) Transwell assay detecting ESCC cell invasive ability after circ-LRP6 depletion. **p < 0.01.

We designed two shRNAs against circ-LRP6 junction site to silence circ-LRP6, as shown in Figure 2(a), these shRNAs could markedly reduce circ-LRP6 expression, but had no effect on its linear isoform. We then performed a series of functional assays in TE-1 and EC109 cells. The CCK-8 assay showed that cell viability was significantly decreased after circ-LRP6 knockdown (Figure 2(b)). Likewise, the number of cell clones was evidently reduced in circ-LRP6-silenced group as compared with control group (Figure 2(c)). Besides, circ-LRP6 depletion resulted in a substantial reduction in invasive capacity of LSC cells (Figure 2(d)).

### Circ-LRP6 acts as a sponge of miR-182

**Figure 3.**
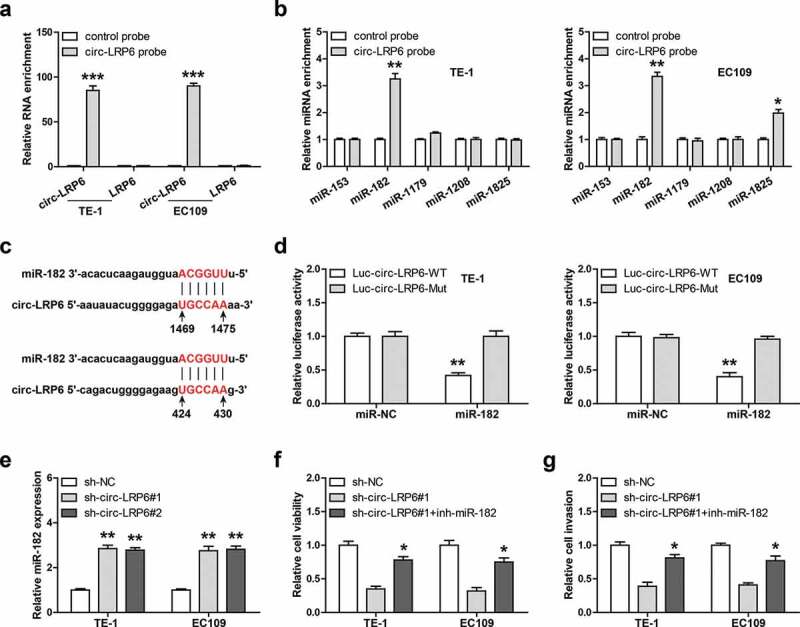
Circ-LRP6 sponges miR-182. (a, b) RNA pull-down assay in two ESCC cells using biotin-labeled circ-LRP6 probe, followed by qRT-PCR analysis. (c) The binding site of miR-182 on circ-LRP6. (d) Luciferase reporter assay in two ESCC cells using wild-type or mutant circ-LRP6 vector. (e) qRT-PCR analysis of miR-182 expression after circ-LRP6 knockdown. (f, g) CCK-8 and Transwell assays testing cell viability and invasion in circ-LRP6-silenced ESCC cells transfected with miR-182 inhibitors. *p < 0.05, **p < 0.01.

Given that circ-LRP6 is mainly located in the cytoplasm, and cytoplasmic circRNAs mainly function as miRNA sponges, we then inferred that circ-LRP6 may function via miRNA. We first designed biotinylated probe against circ-LRP6 junction site to enrich endogenous circ-LRP6, as shown in Figure 3(a), this probe could effectively pull down circ-LRP6 rather then linear LRP6 in both TE-1 and EC109 cells. Through analyzing CircInteractome online tool, we found five miRNAs most likely to bind to circ-LRP6, namely miR-153, miR-182, miR-1179, miR-1208, and miR-1825. Then, we conducted RNA pull-down assay, and the results showed that only miR-182 was abundantly enriched by circ-LRP6 probe in both TE-1 and EC109 cells (Figure 3(b)). There are two miR-182 binding site on circ-LRP6 (Figure 3(c)), we mutated them and performed luciferase reporter assay. As shown in Figure 3(d), overexpression of miR-182 evidently reduced the luciferase activity of wild-type circ-LRP6 luciferase vector, whereas did not affect that of mutant one (Figure 3(d)). Further, miR-182 expression was notably increased in circ-LRP6-depleted ESCC cells in comparison to control cells (Figure 3(e)). Functionally, silencing of miR-182 could significantly rescue the attenuated cell viability (Figure 3(f)) and invasion (Figure 3(g)) caused by circ-LRP6 knockdown in both TE-1 and EC109 cells.

### Myc is the downstream target of circ-LRP6/miR-182 axis

**Figure 4.**
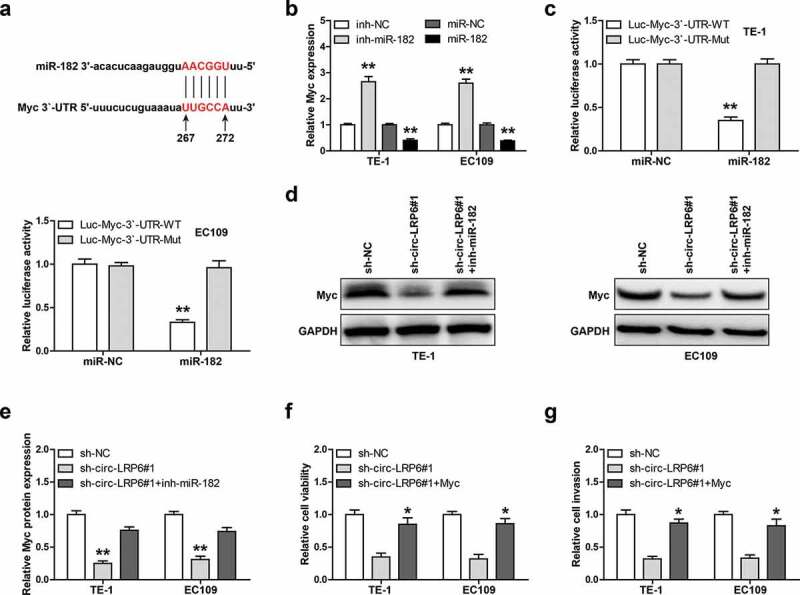
Circ-LRP6 regulates the miR-182/Myc axis. (a) The binding site of miR-182 on Myc 3`-UTR. (b) qRT-PCR analysis of Myc expression after overexpression or silencing of miR-182. (c) Luciferase reporter assay in two ESCC cells using wild-type or mutant Myc 3`-UTR vector. (d, e) Western blot analyzing Myc protein level in circ-LRP6-silenced ESCC cells transfected with miR-182 inhibitors. (f, g) CCK-8 and Transwell assays testing cell viability and invasion in circ-LRP6-silenced ESCC cells transfected with Myc expression vector. *p < 0.05, **p < 0.01.

Through analyzing miRwalk online software, we found that miR-182 may bind to 267 ~ 272 region on Myc 3`-UTR (Figure 4(a)). The qRT-PCR results showed that Myc mRNA level was significantly increased by miR-182 knockdown, while decreased by miR-182 overexpression in these two ESCC cells (Figure 4(b)). Then, we mutated miR-182 binding site and conducted luciferase reporter assay, as shown in Figure 4(c), miR-182 overexpression evidently reduced the luciferase activity of wild-type Myc 3`-UTR luciferase vector, whereas had no effect on that of mutant vector. Importantly, Myc protein expression was dramatically decreased in circ-LRP6-depleted TE-1 and EC109 cells compared to control cells, and this effect was significantly blocked after silencing of miR-182 (Figure 4(d,e)). Functionally, exogenous Myc expression could effectively rescue the weakened cell viability (Figure 4(f)) and invasion (Figure 4(g)) induced by circ-LRP6 knockdown.

### *Knockdown of circ-LRP6 retards tumorigenesis* in vivo

**Figure 5.**
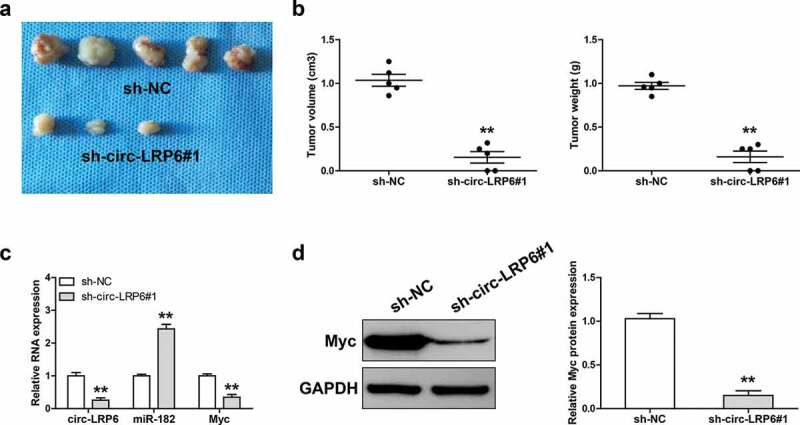
Knockdown of circ-LRP6 inhibits tumor growth. (a) The image showing the tumors of control and circ-LRP6-silenced groups. (b) Tumor volume and weight of control and circ-LRP6-silenced groups. (c) qRT-PCR analysis of circ-LRP6, miR-182, and Myc expression in control and circ-LRP6-silenced groups. (d) Western blot analyzing Myc protein level in control and circ-LRP6-silenced groups. **p < 0.01.

Lastly, we tested the effect of circ-LRP6 *in vivo* by establishing xenograft tumor model. As shown in Figure 5(a,b), the tumor volume and weight of circ-LRP6 knockdown group were significantly smaller than that of control group. The qRT-PCR results displayed that depletion of circ-LRP6 led to a significant increase in miR-182 expression, while a significant decrease in Myc mRNA expression (Figure 5(c)). Consistently, Myc protein level was also dramatically decreased in circ-LRP6-silenced group in comparison to control group (Figure 5(d)).

## Discussion

Although recent studies have confirmed the importance of circ-LRP6 in human diseases, its role in ESCC is still unknown. In the present study, we for the first time investigated the biological function and clinical implication of circ-LRP6 in ESCC. We found that circ-LRP6 was frequently overexpressed in ESCC tissues and linked to aggressive clinical features and dismal prognosis. Circ-LRP6 was identified as a cytoplasmic circRNA that could sponge miR-182 and alleviate the repressive effect of miR-182 on Myc mRNA, resulting in increasing Myc expression, thereby facilitating ESCC development and progression. Importantly, we also conformed the existence of circ-LRP6/miR-182/Myc axis *in vivo*. Therefore, our data suggest that circ-LRP6 is a critical regulator in ESCC carcinogenesis, it will be of great interest to explore its role in other malignant tumors.

Recently, research interest has been reignited in circRNA field. Up to now, more than 100,000 circRNA have been identified, and some of them function as key oncogenes or tumor suppressors in human cancer through regulating different signaling pathway [13]. The main function of circRNA is to act as a miRNA molecular sponge, in which circRNA indirectly affect gene expression via directly absorbing miRNA [14]. The most typical example is CDR1as, it is able to concurrently sponge more than 70 miR-7, acting as a miR-7 antagonist with a miRNA-binding capacity ten times higher than any other known transcript [15,16]. Emerging evidence shows that circRNA is frequently aberrantly expressed in tumor tissues, and participates in tumorigenesis and progression via miRNA [17]. For instance, circ-0001361 was recently found to be highly expressed in bladder cancer, and it could directly interact with miR-491-5p to elevate MMP9, thereby promoting bladder cancer invasion and metastasis [18]. Similarly, circ-CMPK1 was significantly downregulated in nonsmall cell lung cancer and promoted tumor growth by increasing the expression of cyclin D1 via sponging and inhibiting miR-302 activity [19].

In this study, through performing RNA pull-down and luciferase reporter assays, we identified miR-182 as the downstream of circ-LRP6 in ESCC cells. Circ-LRP6 could directly bind to miR-182 to increase Myc expression both *in vitro* and *in vivo*. Myc is a well-known proto-oncogene that is notably overexpressed in human cancer, including ESCC [20,21]. Myc promotes cancer occurrence, development and progression through affecting various oncogenic signaling pathways, such as Wnt/β-Catenin pathway [22]. Its expression level is strictly controlled at the transcriptional and translational levels, and our data provide evidence that Myc is regulated by circRNA at the post-transcriptional level. Importantly, the downregulation of Myc induced by circ-LRP6 knockdown was significantly abolished by silencing of miR-182, and either overexpression of Myc or knockdown of miR-182 could rescued the attenuated malignant phenotype caused by circ-LRP6 depletion, suggesting that the circ-LRP6/miR-182/Myc axis does exist and function in ESCC. Of note, Myc is also a pivotal transcription factor that affects numerous gene expression by directly binding to gene promoter regions [23]. Through analyzing JASPAR database, we found that there are three Myc binding motifs on circ-LRP6 promoter, whether circ-LRP6 is in turn regulated by Myc, thus forming a regulatory loop, needs further in-depth investigation.

## Conclusion

Our results clearly indicate that circ-LRP6 functions as an oncogenic circRNA in ESCC by regulating miR-182/Myc axis, which provide a theoretical basis for it as a potential target of ESCC therapy.

## Highlights

1. Circ-LRP6 is notably increased in ESCC and can be used as a prognostic biomarker.
2. Knockdown of circ-LRP6 inhibits tumor growth *in vitro* and *in vivo*.
3. Circ-LRP6 acts as a sponge of miR-182 in ESCC cells.
4. Dysregulation of circ-LRP6/miR-182/Myc axis contributes to ESCC progression.

## References

[1] Bray F, Ferlay J, Soerjomataram I, et al. Global cancer statistics 2018: GLOBOCAN estimates of incidence and mortality worldwide for 36 cancers in 185 countries. CA Cancer J Clin. 2018;68(6):394–424. 30207593 10.3322/caac.21492

[2] Watanabe M, Otake R, Kozuki R, et al. Recent progress in multidisciplinary treatment for patients with esophageal cancer. Surg Today. 2020;50(1):12–20. 31535225 10.1007/s00595-019-01878-7 PMC6952324

[3] Smyth EC, Lagergren J, Fitzgerald RC, et al. Oesophageal cancer. Nat Rev Dis Primers. 2017;3:17048. 28748917 10.1038/nrdp.2017.48 PMC6168059

[4] Wilusz JE. A 360 degrees view of circular RNAs: from biogenesis to functions. Wiley Interdiscip Rev RNA. 2018;9(4):e1478. 29655315 10.1002/wrna.1478 PMC6002912

[5] Jeck WR, Sorrentino JA, Wang K, et al. Circular RNAs are abundant, conserved, and associated with ALU repeats. Rna. 2013;19(2):141–157. 23249747 10.1261/rna.035667.112 PMC3543092

[6] Rybak-Wolf A, Stottmeister C, Glazar P, et al. Circular RNAs in the mammalian brain are highly abundant, conserved, and dynamically expressed. Mol Cell. 2015;58(5):870–885. 25921068 10.1016/j.molcel.2015.03.027

[7] Li J, Li H, Lv X, et al. Diagnostic performance of circular RNAs in human cancers: A systematic review and meta-analysis. Mol Genet Genomic Med. 2019;7(7):e749. 10.1002/mgg3.749 PMC6625099 31106993

[8] Ng WL, Mohd MT, Shukla K. Functional role of circular RNAs in cancer development and progression. Rna Biol. 2018;15(8):995–1005. 29954251 10.1080/15476286.2018.1486659 PMC6259826

[9] Kristensen LS, Hansen TB, Veno MT, et al. Circular RNAs in cancer: opportunities and challenges in the field. Oncogene. 2018;37(5):555–565. 28991235 10.1038/onc.2017.361 PMC5799710

[10] Hall IF, Climent M, Quintavalle M, et al. Circ_Lrp6, a circular RNA enriched in vascular smooth muscle cells, acts as a sponge regulating miRNA-145 function. Circ Res. 2019;124(4):498–510. 30582454 10.1161/CIRCRESAHA.118.314240

[11] Xue J, Chen C, Luo F, et al. CircLRP6 regulation of ZEB1 via miR-455 is involved in the epithelial-mesenchymal transition during arsenite-induced malignant transformation of human keratinocytes. Toxicol Sci. 2018;162(2):450–461. 29216394 10.1093/toxsci/kfx269

[12] Zheng S, Qian Z, Jiang F, et al. CircRNA LRP6 promotes the development of osteosarcoma via negatively regulating KLF2 and APC levels. Am J Transl Res. 2019;11(7):4126–4138. 31396323 PMC6684910

[13] Guo JU, Agarwal V, Guo H, et al. Expanded identification and characterization of mammalian circular RNAs. Genome Biol. 2014;15(7):409. 25070500 10.1186/s13059-014-0409-z PMC4165365

[14] Verduci L, Strano S, Yarden Y, et al. The circRNA-microRNA code: emerging implications for cancer diagnosis and treatment. Mol Oncol. 2019 Apr;13(4):669–680. 10.1002/1878-0261.12468 PMC6441890 30719845

[15] Memczak S, Jens M, Elefsinioti A, et al. Circular RNAs are a large class of animal RNAs with regulatory potency. Nature. 2013;495(7441):333–338. 23446348 10.1038/nature11928

[16] Hansen TB, Jensen TI, Clausen BH, et al. Natural RNA circles function as efficient microRNA sponges. Nature. 2013;495(7441):384–388. 23446346 10.1038/nature11993

[17] Li J, Sun D, Pu W, et al. Circular RNAs in cancer: biogenesis, function, and clinical significance. Trends Cancer. 2020;6(4):319–336. 32209446 10.1016/j.trecan.2020.01.012

[18] Liu F, Zhang H, Xie F, et al. Hsa_circ_0001361 promotes bladder cancer invasion and metastasis through miR-491-5p/MMP9 axis. Oncogene. 2020;39(8):1696–1709. 31705065 10.1038/s41388-019-1092-z

[19] Cui D, Qian R, Circular LY. RNA circ-CMPK1 contributes to cell proliferation of non-small cell lung cancer by elevating cyclin D1 via sponging miR-302e. Mol Genet Genomic Med. 2020;8(2):e999. 31863641 10.1002/mgg3.999 PMC7005605

[20] Nadal A, Cardesa A. Molecular biology of laryngeal squamous cell carcinoma. Virchows Arch. 2003;442(1):1–7. 12536308 10.1007/s00428-002-0726-6

[21] Allen-Petersen BL, Sears RC. Mission possible: advances in MYC therapeutic targeting in cancer. Biodrugs. 2019;33(5):539–553. 31392631 10.1007/s40259-019-00370-5 PMC6790341

[22] Zhang T, Li N, Sun C, et al. MYC and the unfolded protein response in cancer: synthetic lethal partners in crime? Embo Mol Med. 2020;12(5):e11845. 32310340 10.15252/emmm.201911845 PMC7207169

[23] Dang CV. MYC on the path to cancer. Cell. 2012;149(1):22–35. 22464321 10.1016/j.cell.2012.03.003 PMC3345192

